# Early prostate-specific antigen response post-abiraterone as predictor of overall survival in metastatic castrate-resistant prostate cancer

**DOI:** 10.1186/s12885-019-5729-7

**Published:** 2019-05-31

**Authors:** Joshua P. Schiff, Patrick Cotogno, Allison Feibus, Peter Steinwald, Elisa Ledet, Brian Lewis, Oliver Sartor

**Affiliations:** 1 Tulane Cancer Center, Tulane University School of Medicine, 1430 Tulane Ave., SL-42, New Orleans, LA 70112 USA; 2 Hematology-Oncology Section, Department of Medicine, Tulane University School of Medicine, 1430 Tulane Ave., New Orleans, LA 70112 USA

**Keywords:** mCRPC, PSA, Abiraterone, Response, Survival

## Abstract

**Background:**

Abiraterone is an important agent in the treatment of advanced prostate cancer. Early changes in prostate-specific antigen while on abiraterone in patients with metastatic castrate-resistant prostate cancer potentially have financial and health implications for patients. Limited data is available on early prostate-specific antigen change and subsequent survival given phase III trials did not measure prostate-specific antigen changes before 12 weeks.

**Methods:**

A single-center retrospective study was performed. Metastatic castrate-resistant prostate cancer patients treated with abiraterone (without prior enzalutamide) at Tulane Cancer Center were reviewed with a focus on early prostate-specific antigen decline and relationship to overall survival.

**Results:**

A total of 110 patients were analyzed for prostate-specific antigen response of ≥ 30 and > 50% at 4, 8, and 12 weeks. A prostate-specific antigen response of either > 30% or > 50% at 4, 8, or 12 weeks was associated with improved overall survival at all time points except > 50% decline at 8 weeks. Multivariate analysis indicated, for all time points, that early prostate-specific antigen declines were predictive of overall survival. The neutrophil to lymphocyte ratio and docetaxel pretreatment also were predictive in many, but not all, of the multivariate analyses.

**Conclusions:**

A > 30% or > 50% prostate-specific antigen decline at 4, 8, or 12 weeks provides important information regarding subsequent overall survival for patients with metastatic castrate-resistant prostate cancer. While these data require validation with a large, multi-institutional trial, they can provide physicians with information regarding prognosis and the timing of expected outcomes. These data affirms the notion that prostate-specific antigen responses as early as 4 weeks after abiraterone initiation can be used to inform both patients and physicians about metastatic castrate-resistant prostate cancer outcomes after initiating treatment with this important but costly therapeutic choice.

## Background

Abiraterone and enzalutamide are two of the most important agents introduced in recent years for the treatment of advanced prostate cancer. Abiraterone has been approved by regulatory agencies after pivotal trials in the post-docetaxel metastatic castrate-resistant prostate cancer (mCRPC) space [1], the chemotherapy naïve mCRPC space [2], and the high-risk castrate-sensitive prostate cancer (CSPC) setting [3, 4]. Enzalutamide is approved similarly to abiraterone in the mCRPC space [5, 6], but it also approved in the non-metastatic CRPC (nmCRPC) space [7]. One additional hormonal agent, apalutamide, is approved for nmCRPC [8].

In the post-docetaxel CPRC studies for both abiraterone and enzalutamide, overall survival (OS) was the primary endpoint. For the chemotherapy naïve mCRPC studies, radiographic progression-free survival (rPFS) and OS were co-primary endpoints for both abiraterone and enzalutamide. In all of these cited studies, prostate-specific antigens (PSAs), bone scans, and CT scans were obtained at prescribed intervals. In all the mCRPC studies, PSA testing was obtained at baseline and the first post-treatment PSA was scheduled at twelve weeks post-treatment initiation. Thus early changes in PSA were not available in the large phase III studies with either abiraterone or enzalutamide.

To examine the early changes in PSA, and how they might relate to more important outcomes such as survival, there are limited data. Early changes are potentially important for a variety of reasons, one of the most being that out of pocket costs for patients can be problematic and understanding the probability of longer term benefit is critical for some patients who need to balance the potential benefits of therapy against the costs of therapy. Cost concerns have frequently been voiced about cancer treatments. Though such issues are often discussed in the context of lesser developed countries where medical insurance rarely covers the costs of expensive therapies [9], even insured patients can be overwhelmed by the cost of cancer care [10].

One single institution study at the Royal Marsden specifically looking at early PSA response after abiraterone was published by Rescigno et al. [11]. In that retrospective study (*N* = 274), a > 30% PSA decline at four weeks was associated with longer OS (25.8 vs 15.1 months; hazard ratio (HR) =0.47, *p* < 0.001), in both univariate and multivariate models. Facchini et al. [12] examined PSA declines fifteen days after starting abiraterone and concluded that > 50% declines in PSA were associated with OS (HR = 0.21, *p* < 0.01), however median OS values were not obtainable due to relative immaturity of the data.

Herein we retrospectively examine mCRPC patients treated from a single institution with abiraterone. We focus on early PSA changes predictive of OS in a mature data set. Such information can potentially be helpful to both patients and physician alike.

## Methods

Patients with confirmed metastatic castrate resistant prostate cancer (mCRPC) treated with first line abiraterone between 2012 and 2018 at the Tulane Cancer Center were considered for analysis. PSA value at baseline and at four, eight, and twelve weeks were required for study inclusion. Demographics and baseline characteristics such as prior docetaxel exposure were recorded, as well as routine lab studies including PSA, albumin (Alb), alkaline phosphatase (ALP), neutrophil to lymphocyte ratio (NL ratio), and hemoglobin (Hgb) at start of treatment, when available.

The primary investigation of interest was PSA decline as a predictor of overall PSA response and survival. PSA was procured at four, eight, and twelve. PSA response was defined as > 30% decrease from the PSA at the start of treatment. PSA responses > 50% decrease from the PSA at the start of treatment were also analyzed.

Kaplan-Meier analyses were used to estimate overall survival differences between groups at each time point. Associations between PSA response and survival were evaluated via univariate Cox regression analysis for ≥ 30% and ≥ 50% response at four, eight, and twelve weeks and with multivariate Cox regression models for ≥ 30 and > 50% response at four, eight, and twelve weeks. The variables included in univariate and multivariate regression analysis included PSA responses at four, eight, and twelve weeks and baseline values for PSA, Alb, Hgb, ALP, NL ratio, and prior docetaxel use. Variables with a non-normal distribution were log-transformed (including baseline PSA, ALP, and NL ratio). The models were checked for both proportional hazards and non-collinearity. Multiple imputation was performed on the baseline covariates with missing values, which were deemed missing at random. Missing data and frequency included Alb (35.4%), ALP (26.0%), NL ratio (7.1%), and Hgb (36.28%). Ten iterations were performed and assessed to ensure procedure performance. Additionally, 100 imputations were performed to verify the initial results. Data analysis was completed via SAS/STAT® software. Correlation between various time points was assessed utilizing Spearman’s rank correlation coefficients.

## Results

In this single-institution retrospective review, 159 mCRPC patients were considered for analyses. Ultimately, 110 patients were considered for statistical review, strictly based on data availability. Three subjects were eliminated from the analysis as a result of missing baseline values. Baseline characteristics of patients are summarized in Table 1.

**Table 1:** Baseline characteristics

Characteristics	N	Median (Range)
Total patients	110	
Age	110	74.5 (53–94)
Patients without prior docetaxel exposure	67	
Patients with prior docetaxel exposure	43	
Prostate-specific antigen μg/ml	110	42.3 (2.5–3810)
Alkaline Phosphate U/l	87	101 (9.1–1600)
Albumin g/l	73	3.8 (2.5–4.8)
Hemoglobin g/l	72	12.4 (8.4–15)
Neutrophil to Lymphocyte Ratio	105	3.6 (0.7–18)

Spearman’s rank correlation coefficients were utilized to determine correlation between PSA response at four, eight, and twelve weeks. There was a strong correlation between PSA response at four and eight weeks (Rho = 0.91, *p* = < 0.001), PSA response at four and twelve weeks (Rho = 0.75, *p* = < 0.001), and PSA response at eight and twelve weeks (Rho = 0.89, *p* = < 0.001).

**Fig. 1:**
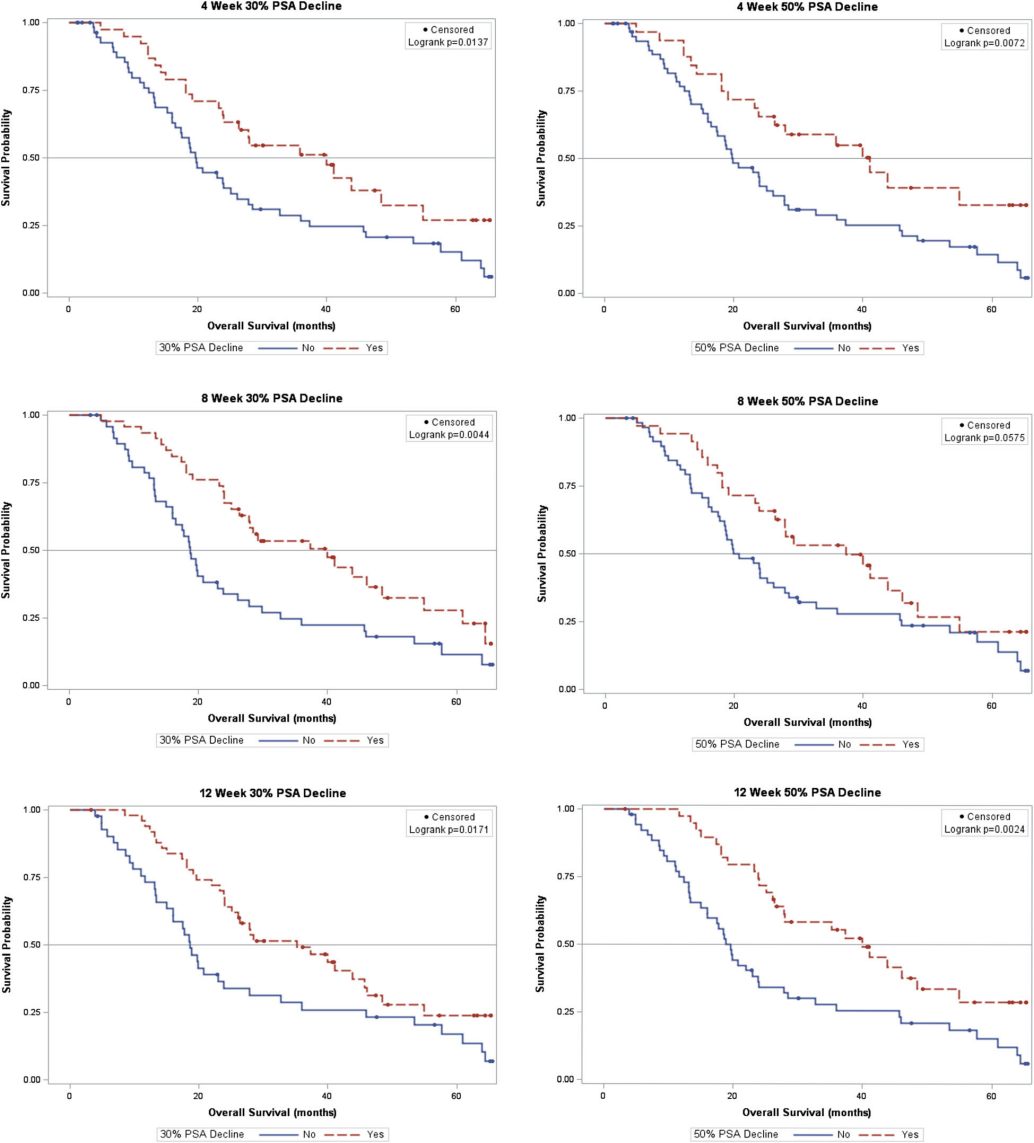
Overall survival. Kaplan Meyer curves for PSA decline ≥ 30% and ≥ 50% at four, eight, and twelve weeks. In order, ≥30% PSA decline at four weeks (top left), ≥50% PSA decline at four weeks (top right), ≥30% PSA decline at eight weeks (middle left), ≥50% PSA decline at eight weeks (middle right), ≥30% PSA decline at twelve weeks (bottom left), and ≥ 50% PSA decline at twelve weeks (bottom right)

A PSA response of ≥ 30% at four, eight, and twelve weeks (HR = 0.54, 0.51, and 0.56, respectively, all *p* < 0.05) was associated with improved OS as compared to subjects without such a decline (see Fig. 1 and Table 2). At four weeks the OS for patients with a PSA response ≥ 30% was significantly greater than patients without a ≥ 30% PSA response, and this was consistent at all the time points (Table 2). For those with a ≥ 30% PSA response at the four, eight, and twelve week time points, OS varied from 35.2 months to 40.0 months with the longest survivals for those with the declines at four and eight weeks. For those with a ≥ 50% PSA response at the four, eight, and twelve weeks points, OS varied from 37.3 months to 41.1 months with the longest survivals for those with the declines at four weeks. Confidence intervals for OS overlapped for those with > 30% or > 50% declines at all the time points evaluated.

**Table 2:** Overall survival data

Outcome (n, %)	Survival (months)	*p*-value	HR (95% CI)	*p*-value
≥30% PSA response at 4 weeks (97)				
Yes (38, 39.2)	40.0 (23.9–55.0)	0.014	0.54 (0.33–0.89)	**0.015**
No (59, 60.8)	19.7 (16.0–25.1)			
≥50% PSA response at 4 weeks (97)				
Yes (32, 33.0)	41.1 (23.2-NE)	0.007	0.49 (0.28–0.83)	**0.006**
No (65, 67.0)	19.9 (16.0–26.1)			
≥30% PSA response at 8 weeks (95)				
Yes (46, 48.4)	40.0 (25.1–48.4)	0.004	0.51 (0.32–0.82)	**0.005**
No (49, 51.6)	18.7 (16.0–23.9)			
≥50% PSA response at 8 weeks (95)				
Yes (35, 36.8)	37.4 (23.9–46.0)	0.058	0.62 (0.38–1.02)	0.054
No (60, 63.2)	20.3 (17.8–26.1)			
≥30% PSA response at 12 weeks (93)				
Yes (51, 54.8)	35.2 (24.0–45.7)	0.017	0.56 (0.35–0.91)	**0.019**
No (42, 45.2)	18.6 (13.4–23.9)			
≥50% PSA response at 12 weeks (93)				
Yes (40, 43.0)	40.0 (26.1–55.0)	0.002	0.47 (0.29–0.77)	**0.002**
No (54, 57.0)	19.6 (15.0–23.9)			

Data from Kaplan Meyer and univariate cox regression analyses for a PSA response ≥30% and ≥ 50% at four, eight, and twelve weeks. Median survival is presented in months (95% CI). NE = not evaluable. *P*-values in bold text are statistically significant (*p* ≤ 0.05)

Multivariable Cox regression analysis was then utilized to determine which variables remained significant for OS taking baseline Alb, Hgb, Alp, PSA, NL ratio, prior docetaxel use and PSA declines at four weeks into account. A ≥ 30% PSA response at four weeks was associated with improved OS (HR = 0.51, 95% CI 0.29–0.90, *p* = 0.021) (Table 3). In addition baseline NL ratio and prior docetaxel use were also significant. Cox multivariate regression analysis was additionally performed for a ≥ 30% PSA response at eight and twelve weeks, with both being associated with a statistically significant increase in rate of survival. For the > 30% PSA decline at eight weeks HR = 0.40, 95% CI 0.24–0.69, *p* = 0.001, and at twelve weeks, HR = 0.48, 95% CI 0.27–0.83, *p* = 0.009. Assessments of additional variables in the multivariate analyses for eight and twelve weeks were similar except that prior docetaxel was not a significant predictor of OS at eight or twelve weeks. We also performed (not shown) Cox multivariable regression analyses at all time points for > 50% PSA decline. Consistent significance was found for the > 50% PSA declines at all time points examined (*p* = 0.019, *p* = 0.032, and *p* = 0.002 at four, eight, and twelve weeks). Baseline NL ratio was also significant at all the time points. Baseline PSA and prior docetaxel were inconsistently significant. Post-abiraterone treatment data was not fully accessible due to referral patterns.

**Table 3:** Cox multivariate regression analysis

Time Point	Variable	HR (95% CI)	*p*-value
4 Weeks (*n* = 93)	≥30% PSA response at 4 weeks	0.51 (0.29–0.90)	**0.021**
	Alb	0.99 (0.51–1.94)	0.978
	Hgb	0.90 (0.73–1.10)	0.306
	ALP	1.47 (0.91–2.35)	0.112
	PSA	1.13 (0.93–1.37)	0.205
	NL Ratio	1.72 (1.13–2.62)	**0.012**
	Prior docetaxel use	0.47 (0.27–0.80)	**0.005**
8 Weeks (*n* = 92)	≥30% PSA response at 8 weeks	0.40 (0.24–0.69)	**0.001**
	Alb	1.14 (0.56–2.30)	0.716
	Hgb	0.96 (0.80–1.15)	0.645
	ALP	1.50 (0.95–2.34)	0.079
	PSA	1.30 (1.07–1.58)	**0.010**
	NL Ratio	1.80 (1.20–2.70)	**0.005**
	Prior docetaxel use	0.75 (0.44–1.28)	0.285
12 Weeks (*n* = 91)	≥30% PSA response at 12 weeks	0.48 (0.27–0.83)	**0.009**
	Alb	0.94 (0.50–1.78)	0.849
	Hgb	0.94 (0.78–1.13)	0.499
	ALP	1.46 (0.90–2.37)	0.122
	PSA	1.19 (1.00–1.44)	0.083
	NL Ratio	1.87 (1.22–2.87)	**0.004**
	Prior docetaxel use	0.65 (0.38–1.10)	0.108

Cox multivariate regression analyses presented for a ≥ 30% response at four, eight, and twelve weeks along with the variables incorporated into the analyses. *P*-values in bold text are statistically significant (*p* ≤ 0.05)

## Discussion

Our data confirms the previous findings of the prior Rescigno et al. [11] retrospective study at the Royal Marsden showing the importance of four week PSA changes in predicting OS. Unlike the Rescigno et al. study, this study evaluated ≥ 30% and ≥ 50% PSA response evaluated as a predictor of OS overall survival at all early time points (four, eight, and twelve weeks). Though there were minor differences in HR and statistical significance, all confidence intervals overlapped and these data do not support meaningful differences between 30% or 50% PSA declines at four, eight, and twelve weeks when predicting eventual OS.

NL ratio has previously been shown to have an impact on PSA response and OS after abiraterone [13] and our analyses indicate that the OS is influenced by this variable as well. Thus our NL ratio data are consistent with prior literature.

Based on these data, the recommendation to the physician is not to stop treatment at four weeks. However, these data can provide both the treating physician and the patient with critical information with regards to prognosis. One can utilize this platform to establish prognosis, to a statistically significant degree, by a simple-metric that is easily measured. Furthermore, it is critical to mention that the decision to withdraw or continue treatment cannot be solely based on this metric, but that other factors such as cost, quality of life, and insurance coverage, amongst others, must be incorporated into the clinical action plan.

This study has a number of limitations. This was a single-institution retrospective study, and therefore the results are potentially biased in both location and time. There is certainly a need to validate these findings with independent, multi-institutional datasets. Furthermore, not all abiraterone treated patients were included as variables necessary for these analyses were sometimes missing. Furthermore, multiple imputation was utilized to complete the datasets for the multivariate analyses. Some of the variables of known prognostic importance such as ECOG performance status and LDH were not complete in this dataset and analyses including these variables were not performed.

## Conclusions

These data suggest that clinicians and patients alike can utilize early PSA response criteria to inform the probability of subsequent events. These types of data are lacking when viewing the large phase III trials in mCRPC with abiraterone [1–4], which delayed assessment of PSA until twelve weeks after starting therapy. Though the difference between four and twelve weeks may seem minor, the cost of abiraterone for eight weeks might exceed $16,000, which is a substantial. Using these data, and the PSA data from the Royal Marsden at four weeks, patients can be better informed of their prognosis after only four weeks of abiraterone exposure and be better empowered to make decisions regarding their health care expenditures.

## Abbreviations

Alb: Albumin
ALP: Alkaline phosphatase
CSPC: Castrate-sensitive prostate cancer
Hgb: Hemoglobin
HR: Hazard ratio
mCRPC: Metastatic castrate-resistant prostate cancer
NL ratio: Neutrophil to lymphocyte ratio
nmCRPC: non-metastatic castrate-resistant prostate cancer
OS: Overall survival
PSA: Prostate-specific antigen
rPFS: Radiographic progression-free survival
